# Evolutionary history of enigmatic bears in the Tibetan Plateau–Himalaya region and the identity of the yeti

**DOI:** 10.1098/rspb.2017.1804

**Published:** 2017-11-29

**Authors:** Tianying Lan, Stephanie Gill, Eva Bellemain, Richard Bischof, Muhammad Ali Nawaz, Charlotte Lindqvist

**Affiliations:** 1 Department of Biological Sciences, University at Buffalo (SUNY), Buffalo, NY 14260, USA; 2 SPYGEN, Savoie Technolac - BP 274, Le Bourget-du-Lac Cedex 73375, France; 3 Department of Ecology and Natural Resource Management, Norwegian University of Life Sciences, PO Box 5003, 1432 Ås, Norway; 4 Department of Animal Sciences, Quaid-i-Azam University, Islamabad, Pakistan; 5 Snow Leopard Trust, 4649 Sunnyside Ave N, Suite 325, Seattle, WA 98103, USA; 6 School of Biological Sciences, Nanyang Technological University, Singapore 637551

**Keywords:** Himalaya, mitochondrial DNA, phylogenetics, Tibetan Plateau, *Ursus arctos*, *Ursus thibetanus*

## Abstract

Although anecdotally associated with local bears (*Ursus arctos* and *U. thibetanus*), the exact identity of ‘hominid’-like creatures important to folklore and mythology in the Tibetan Plateau–Himalaya region is still surrounded by mystery. Recently, two purported yeti samples from the Himalayas showed genetic affinity with an ancient polar bear, suggesting they may be from previously unrecognized, possibly hybrid, bear species, but this preliminary finding has been under question. We conducted a comprehensive genetic survey of field-collected and museum specimens to explore their identity and ultimately infer the evolutionary history of bears in the region. Phylogenetic analyses of mitochondrial DNA sequences determined clade affinities of the purported yeti samples in this study, strongly supporting the biological basis of the yeti legend to be local, extant bears. Complete mitochondrial genomes were assembled for Himalayan brown bear (*U. a. isabellinus*) and black bear (*U. t. laniger*) for the first time. Our results demonstrate that the Himalayan brown bear is one of the first-branching clades within the brown bear lineage, while Tibetan brown bears diverged much later. The estimated times of divergence of the Tibetan Plateau and Himalayan bear lineages overlap with Middle to Late Pleistocene glaciation events, suggesting that extant bears in the region are likely descendants of populations that survived in local refugia during the Pleistocene glaciations.

## 1. Introduction

The Tibetan Plateau, the most extensive and highest plateau in the world with an average altitude of 4500 m above sea level, is partly surrounded by the Himalayan range and many of Earth's highest mountains. Dramatic environmental changes caused by the uplift of the plateau and climatic oscillations during the Quaternary glaciations substantially impacted the evolution, diversification, and distribution of local plant and animal species [1]. Because of its heterogeneous habitat and topography, the region sustains a distinct biome with rich biological diversity and high level of endemism [2]. Extant plants and animals on the plateau are likely either descendants of relict colonists that migrated from other areas or recently derived endemic species [3–10]. However, the colonization and population expansion history of many species remains poorly understood, despite current and future impacts of climate change and anthropogenic threats to diversity loss.

**Figure 1.**
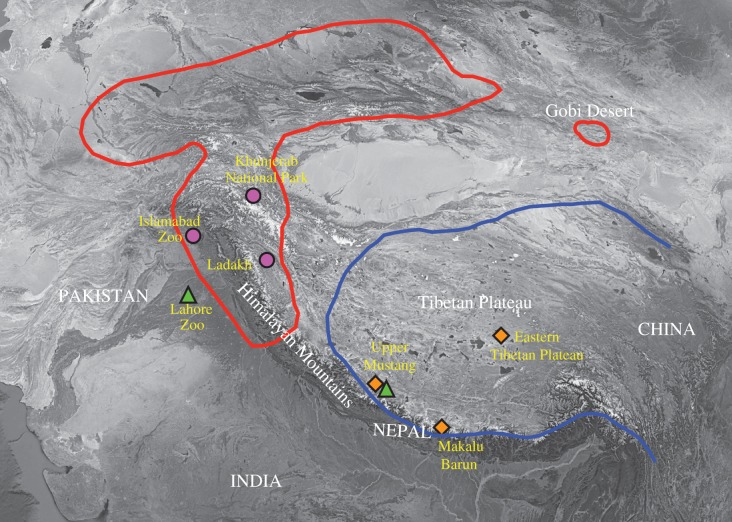
Distribution of Himalayan and Tibetan brown bear and localities of samples studied. Red and blue lines outline the approximate historical range of the Himalayan brown bear and the Tibetan brown bear, respectively (redrawn from Galbreath *et al.* [15]). The triangles, diamonds and circles, respectively, indicate the approximate collecting localities of the studied samples associated with Asian black bear, Tibetan brown bear and Himalayan brown bear.

Two brown bear subspecies, the Himalayan (*Ursus arctos isabellinus*) and the Tibetan (*U. a. pruinosus*) brown bear, inhabit the northwestern Himalayan region and southeastern Tibetan Plateau, respectively [11–14] (figure 1). These two subspecies have distinct skull features and the Himalayan brown bear is characterized by its paler and reddish-brown fur, while the Tibetan brown bear has generally darker fur with a developed, white ‘collar’ around the neck [11]. As the most widely distributed bear in the world, phylogeography of the brown bear has been well studied in North America, Europe and Japan [10,16–24]. However, due to limited sampling, very few studies have been conducted on these enigmatic subspecies. Two complete mitochondrial genomes (mitogenomes) from captive Tibetan brown bears are available, while only two short fragments of mitochondrial DNA (mtDNA) sequences from the Himalayan brown bear have been published [10,15]. Phylogenetic analyses based on these sequences suggested that the Tibetan brown bear might be a relict population of the Eurasian brown bear [10], and that the Himalayan brown bear, which is genetically distinct from the Tibetan brown bear, may represent a more ancient lineage [15]. However, phylogenetic relationships deduced from limited genetic data and number of individuals have put these preliminary findings into question. For example, the phylogenetic placement of a Gobi brown bear (*U. a. gobiensis*) sequence [25] was inconsistent with a later study also including sequences from Himalayan brown bear [15], and phylogenetic trees based on mtDNA control region and cytochrome *b* sequences, respectively, of the Tibetan brown bear were incongruent [26]. The other bear species found to inhabit the Tibetan Plateau–Himalaya region is the Asian black bear (*U. thibetanus*), which historically had a continuous distribution from southeastern Iran through Afghanistan and Pakistan to India, Nepal, China, Korea, Japan, and south into Myanmar and the Malayan peninsula [12,27,28]. Today it occupies a patchy distribution throughout its historic range, including across a narrow band from Pakistan, Kashmir and to Bhutan, the home range of the Himalayan black bear (*U. t. laniger*) [27,29], which was described as distinguished from other black bear populations by its longer, thicker fur and smaller, whiter chest mark [11]. Although the range of Asian black bear overlaps with brown bear in the Tibetan Plateau–Himalaya region, it is mostly found at lower altitudes in forested hills ranging from 1200 to 3300 m [12,29]. So far, little is known about the evolutionary history of black bear in the region and no sequence data are available from the Himalayan black bear. To elucidate the evolutionary and migration history of the Himalayan and Tibetan bears, more genetic data from additional individuals are critically needed.

It has been reported that the brown bear populations in the Tibetan Plateau–Himalaya region have declined by more than half in the past century because of habitat loss, fragmentation, poaching and intense hunting by humans [12,29–31]. Facing the same threats as brown bears, Asian black bear populations have also decreased in the past few decades [29,32,33]. The Himalayan brown bear is listed in the IUCN (International Union for the Conservation of Nature) red list of threatened species as critically endangered [34], while the Asian black bear is listed as vulnerable [27]. Hence, clarifying population structure and genetic diversity for conservation management purposes is also urgently needed for these endangered bear species.

The Tibetan Plateau–Himalaya region is also known for the legend of purported ‘hominid’-like creatures, referred to as the ‘yeti’, ‘chemo’, ‘mheti’ or ‘bharmando’, among other regional monikers (for simplicity they are referred to in this paper as yeti). Despite decades of research and anecdotal association with bears and other mammals in the region [35,36], the species identity of the mysterious yeti is still debated, given the lack of conclusive evidence. A survey of hair samples attributed to yeti and other anomalous, supposed primates, was recently conducted to identify their genetic affinities [37]. Based on a short fragment of the mtDNA 12S rRNA gene from two samples collected in Ladakh, India and Bhutan, respectively, and a 100% match to a sequence recovered from a subfossil polar bear [38], Sykes *et al.* [37] speculated that an unclassified bear species or hybrid of polar bear and brown bear might be present in the Tibetan Plateau–Himalaya region. However, this speculation was critiqued by others [39,40], and their phylogenetic analyses using the sequences from Sykes *et al.* and other available Ursidae sequences did not rule out the possibility that the samples belonged to brown bear. Thus, to get accurate species identification, comprehensive phylogenetic analyses using genetic information from more variable and informative loci are needed.

Here, we report on new analyses of 24 field-collected and museum specimens, including hair, bone, skin and faecal samples, collected from bears or purported yetis in the Tibetan Plateau–Himalaya region. Based on both amplified mtDNA loci as well as complete mitogenomes, we reconstructed maternal phylogenies to increase knowledge about the phylogenetic relationships and evolutionary history of Himalayan and Tibetan bears.

## 2. Material and methods

### (a) Samples

A total of 24 samples, including hair, tissue, bone and faeces, were analysed in this study (electronic supplementary material, table S1). Of these, 12 samples had been collected for a previous analysis of Himalayan brown bear in the Khunjerab National Park, Northern Pakistan [30], two samples were from purported Himalayan brown bears housed in the Lahore and Islamabad Zoos, one bone sample (M-70448) recorded as *U. a. pruinosus* was obtained from the American Museum of Natural History, and nine samples were provided to us by the Reinhold Messner Museum and the Icon Film Company.

### (b) DNA extraction

Genomic DNA from 12 faecal samples collected in the Khunjerab National Park, Northern Pakistan [41], were previously extracted using the QIAmp DNA Stool Kit (Qiagen, USA) in a room dedicated to processing hairs and faeces [30]. DNA from two ethanol-preserved hair samples from Lahore and Islamabad Zoos were isolated in a room dedicated to nucleic acid extraction from modern samples. A DNeasy Blood & Tissue DNA Kit (Qiagen, USA) was used according to the manufacturer's protocol, except for the following modifications to optimize extraction of DNA from hair: 10 strands of hair from each sample were cut into fragments of approximately 0.5 cm with a sterile razor blade. Ethanol was allowed to evaporate (approx. 1 h), and hair fragments were transferred to a microcentrifuge tube. Three hundred microlitres of ATL buffer, 20 µl proteinase K, 20 µl 1M DTT (dithiothreitol) and 4 µl RNase A were added, and samples were incubated at 56°C overnight until completely lysed. A negative control was prepared alongside each hair sample. Following lysis, 300 µl AL buffer and 300 µl 100% EtOH were added to each sample, and the mixture was pipetted into the DNeasy Mini Spin Column and centrifuged for 2 min. DNA was eluted twice with 50 µl AE buffer for a total elution volume of 100 µl. The remaining 10 samples, which had not been intentionally preserved for later extraction of DNA, were regarded as non-modern (ancient) samples, and thus DNA extractions and pre-amplifications were performed in a dedicated state-of-the-art cleanroom facility, physically separated from any modern DNA laboratory and appropriate for ancient DNA research. The following protocols designed for ancient DNA extraction were used: for bone samples, 50–100 mg fine bone powder was obtained from each sample by using a dental drill (HKM Surgical Handpiece, Pearson Dental, USA), and 50–100 mg skin samples were sliced into approximately 1 mm pieces with a sterile razor blade. DNA from the bone powder and the sliced skin samples was extracted using the protocol in Dabney *et al*. [42]. DNA from the hair samples were extracted using the protocol provided by Gilbert *et al*. [43] with the following modifications: 1 ml digestion buffer was used for each hair extraction. After purification with phenol and chloroform, additional purification was performed using Qiagen MinElute PCR Purification Kit (Qiagen, USA). Finally, a 12.5 µl EB buffer elution step was performed twice to obtain a total elution volume of 25 µl. DNA from approximately 100 mg faecal samples was extracted using the QIAmp DNA Stool Kit (Qiagen, USA). The final elution step was also performed twice to obtain a total volume of 100 µl. Negative controls were prepared alongside all extractions.

### (c) PCR amplification

PCR amplifications from modern DNA were performed in a 25 µl reaction volume each containing 2.5 µl of 10 × PCR buffer (Applied Biosystems, USA), 1.0 µl of dNTP mixture (2.5 mM each dNTP; Applied Biosystems), 2.5 µl of MgCl_2_ (25 mM, Applied Biosystems), 0.1 µl of *Taq* DNA polymerase (5–10 U µl^−1^; Applied Biosystems, AmpliTaq Gold), 1 µl each of the forward and reverse primers (10 µM), 2 µl of the genomic DNA and 17.4 µl of H_2_O. The PCR reaction mix for ancient DNAs was prepared in the cleanroom by adding 21 µl H_2_O, 1 µl of each forward and reverse primer, and 2 µl genomic DNA to each GE illustra PuReTaq Ready-To-Go PCR bead (GE Healthcare, USA). A touchdown thermal cycling protocol was used as follows: 10 min at 94°C, 10 cycles of 30 s at 94°C, 30 s annealing with the temperature decreasing every cycle by 0.5°C from 55°C to 50°C, and 30 s extension at 72°C, followed by 25 cycles the annealing temperature set to 50°C and denaturation and extension phases as above. For samples of unknown identity, two sets of mtDNA 12S rRNA primers [44,45] were used to determine their approximate taxonomic affinity. Bear-specific primers targeting the mtDNA control region and cytochrome *b* ([46] and primers designed for this study; see electronic supplementary material, table S2) were used for samples identified as ursid bears. PCR products were Sanger sequenced directly using the same primers as in the PCR.

### (d) Mitochondrial genome target enrichment and sequencing

Fifty microlitres of DNA extracts from four samples were sent to MYcroarray (http://www.mycroarray.com) for preparation of Ion Torrent sequencing libraries and mtDNA target enrichment and sequencing, using the following protocol. Sample libraries were quantified using spectrofluorometry, which indicated between 5 and 255 total nanograms (0.2–8.5 ng µl^−1^) of double-stranded DNA. Each library was then individually target enriched using a custom-designed ursid mitogenome bait set manufactured by MYcroarray. The standard MYbaits v. 3.0 protocol was applied with hybridization for 21 h at 60°C at all relevant steps. Following clean up, half of each bead-bound library was amplified in a 50 µl reaction with universal Ion Torrent adapter-primers for 10 cycles using a KAPA HiFi premix (KAPA Biosystems) and the manufacturer's recommended thermal profile coupled with 62°C annealing temperature. After amplification, the beads were pelleted and the supernatant was purified using SPRI beads and eluted in Tris-HCl buffer containing 0.05% Tween-20. The enriched libraries were quantified with spectrofluorometry, which indicated between 1.12 and 4.21 total nanograms dsDNA per library (0.03–0.12 ng µl^−1^). Equal masses of each library were pooled, bead-templated and sequenced alongside other project libraries on the Ion Proton platform using the Ion PI Chip Kit v2 chemistry. Following sequencing, reads were de-multiplexed, quality trimmed and filtered using the default settings on the Ion Torrent Suite v. 4.4.3.

### (e) Mitochondrial genome assembly

Assembly of mitochondrial genomes was performed using the following strategy: species-specific mitochondrial reference genomes were selected from initial species identification based on phylogenetic analyses of amplicon sequences (results not shown). All Ion Torrent reads were first aligned against the reference genome using BWA *aln* (v. 0.7.13) [47] using the default parameters, except for the parameter ‘-l 1024’ to disable the seed and increase high-quality hits for the damaged ancient DNA reads [48]. The remaining unmapped reads were then aligned against the same reference using BWA *mem* with default parameters (see electronic supplementary material, table S3, for assembly statistics). We filtered for human contamination by applying an edit-distance based strategy [48]. All reads were mapped to a human mitochondrial genome reference (NCBI accession J01415.2) using the same BWA mapping method described above. Reads with a higher mapping edit-distance to human mtDNA than to bear mitochondrial genomes were considered of likely human origin and were removed from the bear mitogenome mapping results. PCR duplicates were removed with the MarkDuplicates tool in the Picard software suite v. 1.112 using lenient validation stringency (http://broadinstitute.github.io/picard/). Consensus calling was carried out using Samtools *mpileup* [49] with default settings.

### (f) Phylogenetic analyses

Complete mitochondrial genomes, partial control region sequences, and cytochrome *b* sequences for 11 Asian black bears, 76 American black bears, two cave bears (*U. spelaeus*), 200 brown bears, and 52 polar bears were obtained from GenBank (electronic supplementary material, table S4). Two GenBank datasets were created: one dataset included only complete mitogenomes for the non-Tibetan/Himalayan bears and both partial (amplicon sequences) and complete mitogenomes for Tibetan and Himalayan bear lineages, while the other dataset included both amplicon sequences and complete mitogenomes for non-Tibetan/Himalayan bears. All new sequences produced in this study were added to these two GenBank datasets and used in the phylogenetic analyses. Sloth bear (*U. ursinus*) and sun bear (*U. malayanus*) sequences were included to root the trees (electronic supplementary material, table S4). Alignments were generated using MAFFT [50] followed by manual adjustment in BioEdit [51] to exclude the variable number tandem repeats of the D-loop. The total length of the final alignment was 16 412 bp. Maximum-likelihood (ML) phylogenetic analyses were performed using RAxML-HPC BlackBox v. 8.2.8 [52] in the CIPRES Science Gateway under the GTR substitution model, which was identified as the best-supported model by jmodeltest2 [53,54]. A total of 1000 bootstrap replicates were conducted to evaluate branch support. Bayesian inference (BI) phylogenetic analyses were carried out using MrBayes v. 3.2.6 [55] in two runs of 5 000 000 Markov chain Monte Carlo (MCMC) generations, with trees for estimation of the posterior probability distribution sampled every 100 generations. The best-fit substitution model was determined by the program by setting Nst=mixed; 500 000 trees were discarded as burn-in.

### (g) Divergence time estimation

Bayesian MCMC-based divergence time estimation was carried out using BEAST version 1.8.0 under the GTR substitution model. The dataset used for molecular dating analysis included only complete mitogenome, since shorter mtDNA regions (e.g. control region and cytochrome *b*) are generally associated with considerable uncertainty and may bias molecular dating analyses due to homoplasy [10,17]. The uncorrelated lognormal relaxed clock and the constant size coalescent prior were used. Radiocarbon dates and stratigraphically estimated dates for four ancient sequences were used to calibrate ages for terminal nodes, including three sequences from extinct bear species (*U. spelaeus* and *U. deningeri*) dated to 31.8 thousand years (ka) BP [56], 44.1 ka BP [57], and 409 ka BP [42], an approximately 120 ka BP polar bear subfossil [38], and seven European brown bears dated to approximately 4.1–37 ka BP [58]. Trees were sampled every 1000 generations from a total of 1 000 000 000 generations. The maximum clade credibility tree was generated using TreeAnnotator, implemented in the BEAST package [59], with 10% burn-in. Effective sampling size value greater than 200 for all parameters sampled from the MCMC and the posterior distributions were examined using Tracer v. 1.6 [60].

## 3. Results

### (a) Identity and phylogenetic placement of the Tibetan Plateau–Himalayan samples

**Figure 2.**
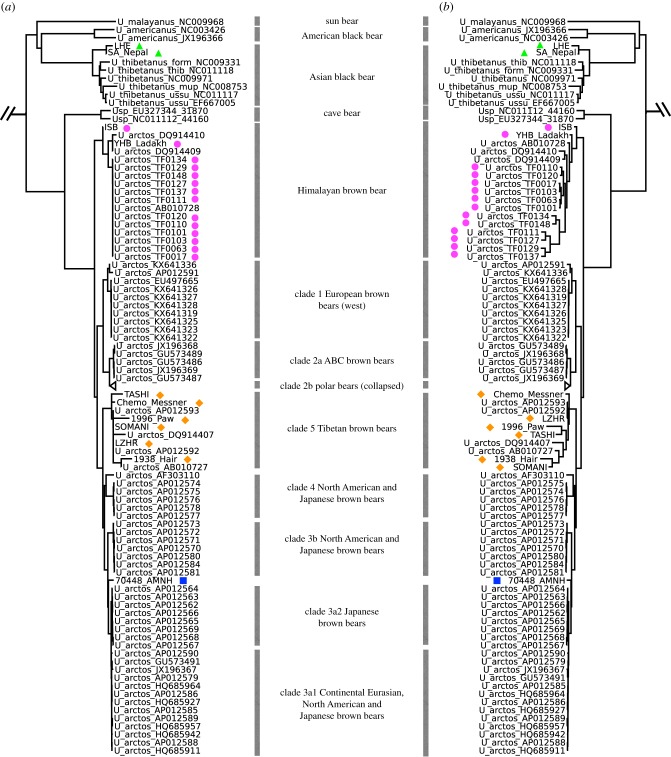
Phylogenetic trees based on (*a*) ML and (*b*) BI analyses of new mtDNA sequence data produced in this study and sequence data obtained from GenBank. New sequences are marked with triangles, diamonds, circles and a square, indicating the Asian black bear, Tibetan brown bear, Himalayan brown bear and the brown bear from the AMNH, respectively. GenBank data include complete mitogenomes of non-Tibetan–Himalayan bears, as well as amplicon and complete mitochondrial sequences of Tibetan and Himalayan bears. Major maternal clades and their geographic range are labelled following [10,17]. See electronic supplementary material, figures S2 and S3, for complete versions of the trees, shown with posterior probability and bootstrap values.

Except for one tooth sample collected from a stuffed exhibit at the Reinhold Messner Mountain Museum, which BLAST-matched dog (*Canis lupus familiaris*), all other samples were identified as ursid bears. ML tree reconstruction based on amplicon and mitogenome sequences (electronic supplementary material, figure S1) grouped the 23 samples within four bear lineages: Himalayan brown bear, Tibetan brown bear, Continental Eurasian brown bear and Asian black bear. Complete mitogenomes were assembled from one individual in each of the four identified bear lineages (electronic supplementary material, table S3). ML and BI phylogenetic trees were reconstructed using the newly obtained amplicon sequences, complete mitogenome sequences, and previously published bear mtDNA sequences, using sloth bear (*U. ursinus*) as an outgroup (figure 2 and electronic supplementary material, figures S2 and S3). In general, the ML and BI tree topologies are consistent and in agreement with previous studies [10,17,61], with all major polar, brown and black bear clades well-resolved and strongly supported. The two Tibetan–Himalayan black bear samples formed a well-supported sister lineage to all other Asian black bear subspecies. The polar and brown bears grouped into nine clades (clades 1, 2a, 2b, 3a1, 3a2, 3b, 4, 5 and a Himalayan clade, with numerical clade nomenclature following [10,17]). Fourteen samples collected in Pakistan and the Himalayas grouped with a previously reported Gobi brown bear (GOBI-1) and two Himalayan brown bears (DQ914409 and DQ914410), and formed a sister lineage to all other brown and polar bear clades with strong bootstrap support. Six samples collected from the Tibetan Plateau grouped with previously sequenced Tibetan brown bears, which together formed a sister clade to several other North American and Eurasian brown bear lineages (clade 3a1, 3a2, 3b and 4). One specimen (M-70448), which was sampled from the American Museum of Natural History's mammal collection and identified as a Tibetan brown bear, possibly of ‘mixed breed’, grouped in clade 3a with brown bears from Syria, Turkey, and animals held at Zoos in Europe [24] (electronic supplementary material, figure S1 and table S4).

### (b) Divergence time estimations

**Figure 3.**
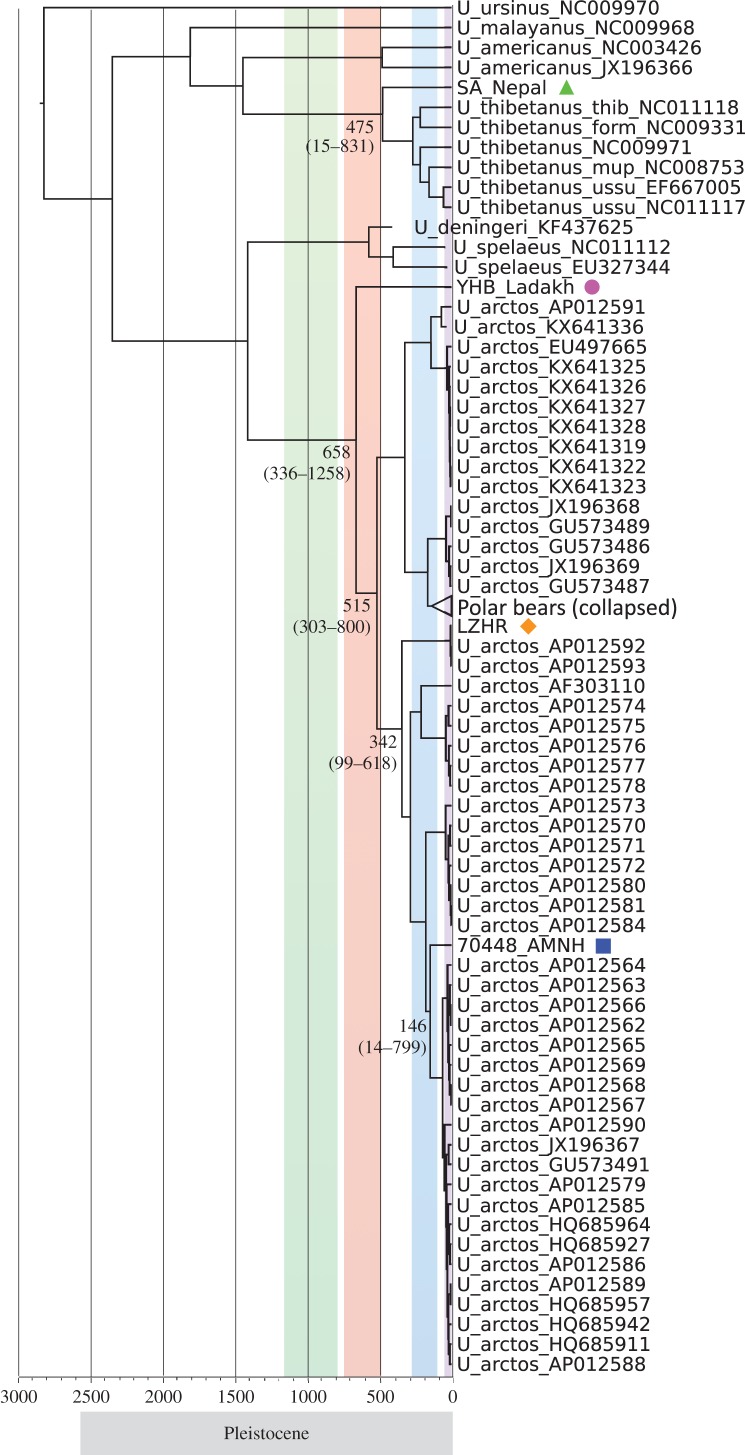
Maximum clade credibility tree from a BEAST analysis based on complete mitogenomes. The numbers at nodes indicate the median estimated divergence time in ka BP (HPD values are shown in brackets and the lower scale indicates time in ka BP). The coloured vertical bars indicate, from left to right, time spans of four Pleistocene glaciations: the Xixabangma, Nyanyaxungla, Guxiang and Baiyu. New mitogenomes sequenced in this study are indicated with symbols as in figure 2. See electronic supplementary material, figure S4, for a complete version of the tree and divergence times estimated for all nodes.

MCMC-based divergence times discussed in the text are shown in figure 3 (see electronic supplementary material, figure S4, for divergence times estimated for all nodes). For the brown bear clades, the divergence time between the Himalayan lineage and all other brown bear lineages was estimated to be 658 ka BP (95% HPD: 336–1258 ka BP). The divergence time between the Tibetan lineage and its sister North American and Eurasian lineages (clade 3 and 4) was estimated at 342 ka BP (95% HPD: 99–618 ka BP), and the split of the Continental Eurasian lineage (clade 3a) was estimated to be 146 ka BP (95% HPD: 14–799 ka BP). For the black bear clades, the ancestor of the Himalayan black bear lineage diverged from other Asian black bear lineages at approximately 475 ka BP (95% HPD: 15–831 ka BP).

## 4. Discussion

### (a) Phylogenetic placement and evolutionary history of Himalayan and Tibetan brown bears

Few genetic studies have been conducted of bears in the Tibetan Plateau and surrounding Himalaya region, and their evolutionary history remains enigmatic. Particularly little is known about the Himalayan brown bear (*U. a. isabellinus*). First, Masuda *et al.* [25] reported a 269 bp mtDNA control region sequence from a Gobi bear collected from the Great Gobi National Park in Mongolia, and suggested it was more closely related to Western European brown bears based on a neighbour-joining phylogenetic analysis. Later, Galbreath *et al.* [15] investigated homologous DNA fragments from two brown bears collected from the Deosai Plains of the western Himalayas. Their analyses demonstrated that the two Himalayan brown bears grouped together with the Gobi bear, confirming a close relationship between these two populations and a clear separation from European and Tibetan brown bears. Our results, providing more data and better resolution, demonstrate that the Himalayan brown bears, including the previously reported Gobi bear and Deosai bears, form a well-supported, sister lineage to all other extant brown bear clades included here. This result strongly supports Himalayan brown bears as a relict population that diverged early from other brown bear populations.

The phylogenetic position of Tibetan brown bears (*U. a. pruinosus*), which form a sister clade to North American and Eurasian brown bears consistent with previous reports [10,17–19,25], indicates that the Tibetan and other Eurasian brown bears, as well as North American brown bears, are all descendants of a common ancestral lineage. It was proposed that the Tibetan brown bears migrated to the Tibetan Plateau from its source population—ancestral Eurasian brown bears—approximately 343 ka BP, and that they remained geographically isolated from this source population thereafter [10]. Our phylogenetic analyses strongly support this migration scenario.

In our study, brown bear samples collected in the northwestern to western Himalayas were all identified as Himalayan brown bear, while the ones collected in the southeastern Himalayas and Tibetan Plateau were all identified as Tibetan brown bear (figure 1). The historical range of the Himalayan brown bear extends from the north and west of the Taklimakan Desert to the western Himalayas, while the historical range of the Tibetan brown bear lies in the Tibetan Plateau and the southeastern Himalayas [15]. While the Tibetan brown bears share a common ancestry with extant North American and Eurasian brown bears, the Himalayan brown bear appears to have originated from an ancient lineage that experienced long isolation in the mountains of central Asia, at least over the last 658 ka. Although the habitats of the two brown bear subspecies are geographically close, the high-altitude peaks of the Himalayan Mountains have likely impeded migration between these populations, and subsequently kept them as genetically distinct lineages.

### (b) Phylogenetic placement and evolutionary history of the Himalayan black bear

The phylogenetic topology of Asian black bears is in agreement with a previous finding [61], except here we also include the rare Himalayan black bear (*U. t. laniger*), which forms a sister lineage to all other Asian black bears. Although sampling is limited, this result indicates that the Himalayan black bear originated from an ancient lineage and experienced long isolation in the Himalayan Mountains, a similar scenario to the divergence of the Himalayan brown bear lineage. However, the divergence time for the Himalayan black bear is younger, estimated at 475 ka BP, suggesting the isolation of Himalayan black bear occurred later than the isolation of the higher-altitude Himalayan brown bear. Reportedly, other described subspecies occur in the region, the Tibetan (*U. t. thibetanus*) and Indochinese (*U. t. mupinensis*) black bear, but whether these subspecies overlap is unclear given no modern revisionary work exists. Our phylogenetic relationships indicate that individuals from the Himalayas are genetically distant from other populations analysed, suggesting that little if any gene flow has occurred between this and other Asian black bear populations. Similar to the brown bear situation, the high mountains may also have separated the habitats of these black bear subspecies, possibly keeping *U. t. laniger* to the western Himalayas, and *U. t. mupinensis* and *U. t. thibetanus* to the east. Analyses of more individuals throughout the region and inclusion of nuclear DNA would be needed, however, to explore if this pattern is restricted to maternal gene flow only.

### (c) Quaternary climatic oscillations and divergence of local bear lineages in the Tibetan Plateau–Himalaya region

The Tibetan Plateau is one of the youngest plateaus on Earth, created by the collision of the Indian subcontinent with the Eurasian continental plate in early Cenozoic times, followed by diachronous and extensive surface uplifts in the Miocene and even into the Pleistocene [62,63]. Although the dates and details of the uplifts have long been debated, many studies indicate they caused dramatic climatic changes and topographic variation, which facilitated the introduction and evolution of new plant and animal clades and greatly influenced the current spatial distribution of local species and their genetic diversity [64]. The Pleistocene glaciations of the Tibetan Plateau, which is closely related to the progressive uplift of the plateau and the surrounding Himalayan Mountains, have been suggested to have had a highly complex pattern, occurring asynchronously with the Northern Hemisphere glaciation events [65]. Four Pleistocene glaciations have been described in several geological and geographical studies [66–68]; the Xixabangma (Early Pleistocene, 1170–800 ka BP), Nyanyaxungla (Middle Pleistocene, 720–500 ka BP), Guxiang (Middle-Late Pleistocene, 300–130 ka BP) and Baiyu (Late Pleistocene, 70–10 ka BP) events. The most widespread Nyanyaxungla glaciation [64,69] was initiated by successive Kunlun-Huanghe tectonic movements. Interestingly, the divergence time of the Himalayan brown bear at around 658 ka BP overlaps with the Nyanyaxungla glaciation event, suggesting that this glaciation event may have caused the initial isolation of Himalayan brown bear. Glacial retreat occurred following the Nyanyaxungla glaciation, causing changes in environmental conditions from cold and arid to warm and wet during the great interglacial period (500–300 ka BP) [68]. Both the divergence of the Himalayan black bear at around 475 ka BP and the Tibetan brown bear at around 342 ka BP overlap with this interglacial period, indicating that ancestors of these bear lineages migrated from lower altitudes to higher altitude locales after glaciers retreated. Subsequently, these populations may have diverged from lower altitude populations due to isolation in the high mountains and the following Guxiang glaciation event. Phylogeographic studies of many Tibetan plant and animal species indicate that local extant plant and animal populations, which mainly derived from colonists migrating from other areas or represent endemic species that diverged recently [3–10], experienced extensive oscillations and survived through glacial periods in multiple refugia or microrefugia on the plateau [1,65,70–75]. Similarly, we speculate that ancestral bear lineages on the Tibetan Plateau and Himalayan Mountains likely immigrated to the region from nearby Asian locales. These ancestral lineages then likely experienced extensive population oscillations caused by local climatic changes and diverged from other bear populations in refugia during the Pleistocene glaciations.

## 5. Conclusion

Samples collected in the field and archived in museum or private collections can significantly aid in our understanding of the genetic variation and phylogeographic patterns of rare and widespread species. To determine accurate species identification and clade affinity, however, phylogenetically informative genetic markers and appropriate phylogenetic analyses are critically needed. Based on a BLAST search using a 104 bp fragment of the mitochondrial 12S rRNA locus, which gave a 100% match to a complete mitogenome recovered from a subfossil polar bear [38], Sykes *et al*. [37] suggested that a previously unrecognized bear species or possibly a hybrid between brown bear and polar bear exists in the Himalayas. However, as also demonstrated by others [39,40], the short 12S rRNA gene fragment is insufficiently informative to determine precise taxonomic identity, particularly among closely related species, although it can be a useful screening marker to assess preliminary species affinities. We isolated DNA and assembled a complete mitogenome from a hair sample (collected in Ladakh, India, and named ‘YHB’ in this study), which based on their shared collection locality and other anecdotal evidence obtained from Icon Films, our sample source, may come from the same specimen that Sykes *et al.* [37] speculated represents an unknown or hybrid bear. Here, we unambiguously show that this sample is from a bear that groups with extant Himalayan brown bear. Similarly, we were able to determine the clade affinities of all other purported yeti samples in this study and infer their well-supported and resolved phylogenetic relationships among extant bears in the Tibetan Plateau and surrounding Himalayan Mountains. This study represents the most rigorous analysis to date of samples suspected to derive from anomalous or mythical ‘hominid’-like creatures, strongly suggesting that the biological basis of the yeti legend is local brown and black bears.

## Supplementary Material

Supplementary Tables S1-S4

## Supplementary Material

Supplementary Figure S1

## Supplementary Material

Supplementary Figure S2

## Supplementary Material

Supplementary Figure S3

## Supplementary Material

Supplementary Figure S4

## Data accessibility

The DNA sequences generated in this study are deposited in the NCBI database under accession nos MG066702–MG066705 and MG131869–MG131905.
