# Supplementary material for: Evolutionary history of enigmatic bears in the Tibetan Plateau–Himalaya region and the identity of the yeti
Source: Proc Biol Sci. 2017 Nov 29;284(1868):20171804. doi: 10.1098/rspb.2017.1804 (PMC5740279; doi:10.1098/rspb.2017.1804)
Supplement: Supplementary Tables S1-S4 [file rspb20171804supp1.docx]

**Electronic Supplementary Material**

Evolutionary history of enigmatic bears in the Tibetan Plateau-Himalaya Region and the identity of the Yeti

Tianying Lan^1^, Stephanie Gill^1^, Eva Bellemain^2^, Richard Bischof^3^, Muhammad Ali Nawaz^4,5^, Charlotte Lindqvist^1,6^

Proceedings of the Royal Society B, DOI: 10.1098/rspb.2017.1804

Supplementary Tables S1-S4

**Table S1.** Collected samples and information on tissue type, species identity, collecting localities, origin, and DNA sequence data.

| **Sample ID** | **Species identity** | **Sample type** | **Collecting location** | **Notes on origin** | **mtDNA data obtained** | **GenBank accession numbers** |
| --- | --- | --- | --- | --- | --- | --- |
| TH1 | *C. lupus familiaris* | tooth | Tibetan Plateau | Tooth from stuffed “chemo” (the Messner Mountain Museum; collected 1938-39). | amplicon | N/A |
| 1938 hair | *U. arctos pruinosus* | hair | Tibetan Plateau | Hair from stuffed “chemo” (the Messner Mountain Museum; collected 1938-39). | amplicons | MG131870, MG131882, MG131901 |
| 1996 paw | *U. arctos pruinosus* | skin | Tibetan Plateau | Dried tissue from a bear paw (the Messner Mountain Museum; collected 1996). | amplicons | MG131871, MG131883 |
| Chemo | *U. arctos pruinosus* | fecal | Eastern Tibetan Plateau | Scat collected from an area of supposed 'chemo' sighting (the Messner Mountain Museum; collection date unknown). | amplicons | MG131874, MG131875, MG131876, MG131887, MG131888, MG131904, MG131905 |
| YHB | *U. arctos isabellinus* | hair | Zanskar Region, Ladakh, India | Mummified animal in a monastery (collected 2014; provided by Harry Marshall). | mitogenome | MG066705 |
| LZHR | *U. arctos pruinosus* | hair | Barun Valley, Mt. Makalu, Sankhuwasabha district, Nepal | So-called Lisanovich Hair collected by a Jesuit priest (collected 1959; provided by Charles Allen). | mitogenome | MG066703 |
| Tashi | *U. arctos pruinosus* | bone | Tibetan Plateau | Piece of femur bone provided by a spiritual healer, Pemba Tashi, who found a decayed body in a cave on the Tibetan Plateau, purportedly of yeti (“mheti”) origin (collected 2008-09). | amplicons | MG131872, MG131885, MG131903 |
| Somani | *U. arctos pruinosus* | hair | Chumjung, Upper Mustang, Nepal | Collected by a nomadic herdsman, Sonam Norbu (collected 2014). | amplicons | MG131884, MG131902 |
| SA | *U. thibetanus* | skin | Tsarang Village, Upper Mustang, Nepal | Dried tissue from a hand/paw, purportedly of yeti (“mheti”) origin, from a monastic relic (collection date unknown). | mitogenome | MG066704 |
| M-70448 | *U. arctos* | bone | Unknown | Specimen at the American Museum of Natural History listed as *U. arctos pruinosus*, possibly of “mixed breed”, received from New York Zoo (collection date unknown). | mitogenome | MG066702 |
| LHE | *U. thibetanus* | hair | Unknown | Hair samples collected by Lahore Zoo staff (collection date unknown). | amplicons | MG131869, MG131881 |
| ISB | *U. arctos isabellinus* | hair | Unknown (Pakistan) | Hair samples from 10-yr old female bear collected by Islamabad Zoo staff (collection date unknown). | amplicons | MG131873, MG131886 |
| TF0017 | *U. arctos isabellinus* | fecal | Khunjerab National Park, N. Pakistan | Scat collected during fieldwork in 2010 | amplicons | MG131877, MG131889 |
| TF0063 | *U. arctos isabellinus* | fecal | Khunjerab National Park, N. Pakistan | Scat collected during fieldwork in 2011 | amplicons | MG131878, MG131890 |
| TF0101 | *U. arctos isabellinus* | fecal | Khunjerab National Park, N. Pakistan | Scat collected during fieldwork in 2011 | amplicons | MG131879, MG131891 |
| TF0103 | *U. arctos isabellinus* | fecal | Khunjerab National Park, N. Pakistan | Scat collected during fieldwork in 2011 | amplicons | MG131880, MG131892 |
| TF0110 | *U. arctos isabellinus* | fecal | Khunjerab National Park, N. Pakistan | Scat collected during fieldwork in 2011 | amplicon | MG131893 |
| TF0111 | *U. arctos isabellinus* | fecal | Khunjerab National Park, N. Pakistan | Scat collected during fieldwork in 2011 | amplicon | MG131895 |
| TF0120 | *U. arctos isabellinus* | fecal | Khunjerab National Park, N. Pakistan | Scat collected during fieldwork in 2011 | amplicon | MG131894 |
| TF0127 | *U. arctos isabellinus* | fecal | Khunjerab National Park, N. Pakistan | Scat collected during fieldwork in 2011 | amplicon | MG131896 |
| TF0129 | *U. arctos isabellinus* | fecal | Khunjerab National Park, N. Pakistan | Scat collected during fieldwork in 2011 | amplicon | MG131897 |
| TF0134 | *U. arctos isabellinus* | fecal | Khunjerab National Park, N. Pakistan | Scat collected during fieldwork in 2011 | amplicon | MG131898 |
| TF0137 | *U. arctos isabellinus* | fecal | Khunjerab National Park, N. Pakistan | Scat collected during fieldwork in 2011 | amplicon | MG131899 |
| TF0148 | *U. arctos isabellinus* | fecal | Khunjerab National Park, N. Pakistan | Scat collected during fieldwork in 2011 | amplicon | MG131900 |

**Table S2.** Primers used in this study.

| **Primer ID** | **Forward primer sequence (5'-3')** | **Reverse primer sequence (5'-3')** | **Amplicon length (bp)** | **Mitogenome region** | **Reference** |
| --- | --- | --- | --- | --- | --- |
| L16164 & H16299 | GCCCCATGCATATAAGCATG | GGAGCGAGAAGAGGTACACGT | 175 | control region | Taberlet & Bouvet 1994 |
| 161 &162 | GCGTGCCCCATGCATATAA | GCTCCCGGACTAAGTGAAAT | 131 | control region | This study |
| 163 & 164 | GCAGACCTACTAACACTAAC | TGGCTTACAAGACCAAGGTAA | 194 | cyt *b* | This study |
| 165 & 164 | CACTAACATGAATYGGAGGACA | TGGCTTACAAGACCAAGGTAA | 194 | cyt *b* | This study |
| 12S1094F & 12S1199R | ACTGGGATTAGATACCCCACTATG | ATCGATTATAGAACAGGCTCCTC | 100 | 12S | Melton & Holland 2007 |
| 12Sa & 12So | CTGGGGATTAGATACCCCACTA | GTCGATTATAGGACAGGTTCCTCTA | 151 | 12S | Rohland et al. 2004 |

**Table S3.** Mitochondrial genome sequencing and assembly statistics.

| **Sample** | **Reads sequenced** | **Reads mapped before PCR duplicates removal** | **Reads mapped after PCR duplicates removal** | **Breadth of coverage** | **Depth of coverage** | **Mitogenome reference NCBI accession** |
| --- | --- | --- | --- | --- | --- | --- |
| YHB | 4,523,703 | 4,238,695 | 67,944 | 100.0% | 391.58 | Uar_AF303110 |
| 70448 | 6,130,816 | 2,717,327 | 41,300 | 100.0% | 337.57 | Uar_AF303110 |
| SA | 2,354,990 | 1,592,020 | 33,177 | 100.0% | 233.71 | Uth_NC_009971 |
| LZHR | 1,120,825 | 998,538 | 33,835 | 99.8% | 232.61 | Uar_AF303110 |

**Table S4.** NCBI accessions used in the phylogenetic analyses.

| **Sequence ID** | **Taxon** | **NCBI Accession Number** | **Sample Age** | **Mitchondrial Region** |
| --- | --- | --- | --- | --- |
| U_ursinus_NC009970 | *Ursus ursinus* | NC_009970 | modern | mitogenome |
| U_malayanus_NC009968 | *Ursus malayanus* | NC_009968 | modern | mitogenome |
| U_thibetanus_form_NC009331 | *Ursus thibetanus formosanus* | NC_009331 | modern | mitogenome |
| U_thibetanus_ussu_NC011117 | *Ursus thibetanus ussuricus* | NC_011117 | modern | mitogenome |
| U_thibetanus_ussu_EF667005 | *Ursus thibetanus ussuricus* | EF667005 | modern | mitogenome |
| U_thibetanus_thib_NC011118 | *Ursus thibetanus thibetanus* | NC_011118 | modern | mitogenome |
| U_thibetanus_mup_NC008753 | *Ursus thibetanus mupinensis* | NC_008753 | modern | mitogenome |
| U_thibetanus_NC009971 | *Ursus thibetanus* | NC_009971 | modern | mitogenome |
| U_thibetanus_japo_AB360915 | *Ursus thibetanus japonicus* | AB360915 | modern | CR |
| U_thibetanus_japo_AB360916 | *Ursus thibetanus japonicus* | AB360916 | modern | CR |
| U_thibetanus_japo_AB360917 | *Ursus thibetanus japonicus* | AB360917 | modern | CR |
| U_thibetanus_japo_AB360919 | *Ursus thibetanus japonicus* | AB360919 | modern | CR |
| U_thibetanus_japo_AB3609215 | *Ursus thibetanus japonicus* | AB360921 | modern | CR |
| U_americanus_JX196366 | *Ursus americanus* | JX196366 | modern | mitogenome |
| U_americanus_NC003426 | *Ursus americanus* | NC003426 | modern | mitogenome |
| U_americanus_AF268271 | *Ursus americanus* | AF21120 | modern | cytB |
| U_americanus_AF268265 | *Ursus americanus* | AF12974 | modern | cytB |
| U_americanus_AF268268 | *Ursus americanus* | AF18536 | modern | cytB |
| U_americanus_AF268267 | *Ursus americanus* | AF18535 | modern | cytB |
| U_americanus_AF268262 | *Ursus americanus* | AF14581 | modern | cytB |
| U_americanus_AF268261 | *Ursus americanus* | AF14582 | modern | cytB |
| U_americanus_AF268259 | *Ursus americanus* | AF16110 | modern | cytB |
| U_americanus_U23556 | *Ursus americanus* | U23556 | modern | cytB |
| U_americanus_X82307 | *Ursus americanus* | X82307 | modern | cytB |
| U_americanus_U34267 | *Ursus americanus* | U34267 | modern | CR+cytB |
| U_americanus_U34265 | *Ursus americanus* | U34265 | modern | CR+cytB |
| U_americanus_U34266 | *Ursus americanus* | U34266 | modern | CR+cytB |
| U_americanus_U34261 | *Ursus americanus* | U34261 | modern | CR+cytB |
| U_americanus_U34264 | *Ursus americanus* | U34264 | modern | CR+cytB |
| U_americanus_U34262 | *Ursus americanus* | U34262 | modern | CR+cytB |
| U_americanus_U34260 | *Ursus americanus* | U34260 | modern | CR+cytB |
| U_americanus_U34263 | *Ursus americanus* | U34263 | modern | CR+cytB |
| U_americanus_FJ619658 | *Ursus americanus* | FJ619658 | modern | CR+cytB |
| U_americanus_AY334366 | *Ursus americanus* | AY334366 | modern | CR+cytB |
| U_americanus_FJ619659 | *Ursus americanus* | FJ619659 | modern | CR+cytB |
| U_americanus_FJ619654 | *Ursus americanus* | FJ619654 | modern | CR+cytB |
| U_americanus_AY334364 | *Ursus americanus* | AY334364 | modern | CR+cytB |
| U_americanus_FJ619656 | *Ursus americanus* | FJ619656 | modern | CR+cytB |
| U_americanus_FJ619657 | *Ursus americanus* | FJ619657 | modern | CR+cytB |
| U_americanus_AF012323 | *Ursus americanus* | AF012323 | modern | CR |
| U_americanus_AF012321 | *Ursus americanus* | AF012321 | modern | CR |
| U_americanus_AF012322 | *Ursus americanus* | AF012322 | modern | CR |
| U_americanus_AF012320 | *Ursus americanus* | AF012320 | modern | CR |
| U_americanus_AF012308 | *Ursus americanus* | AF012308 | modern | CR |
| U_americanus_AF012305 | *Ursus americanus* | AF012305 | modern | CR |
| U_americanus_AF012307 | *Ursus americanus* | AF012307 | modern | CR |
| U_americanus_AF012306 | *Ursus americanus* | AF012306 | modern | CR |
| U_americanus_AF012311 | *Ursus americanus* | AF012311 | modern | CR |
| U_americanus_AF012310 | *Ursus americanus* | AF012310 | modern | CR |
| U_americanus_AF012313 | *Ursus americanus* | AF012313 | modern | CR |
| U_americanus_AF012312 | *Ursus americanus* | AF012312 | modern | CR |
| U_americanus_AF012309 | *Ursus americanus* | AF012309 | modern | CR |
| U_americanus_AF012314 | *Ursus americanus* | AF012314 | modern | CR |
| U_americanus_AF012319 | *Ursus americanus* | AF012319 | modern | CR |
| U_americanus_AF012315 | *Ursus americanus* | AF012315 | modern | CR |
| U_americanus_AF012316 | *Ursus americanus* | AF012316 | modern | CR |
| U_americanus_AF012318 | *Ursus americanus* | AF012318 | modern | CR |
| U_americanus_EF198812 | *Ursus americanus* | EF198812 | modern | CR |
| U_americanus_EF198844 | *Ursus americanus* | EF198844 | modern | CR |
| U_americanus_GU724192 | *Ursus americanus* | GU724192 | modern | CR |
| U_americanus_GU724174 | *Ursus americanus* | GU724174 | modern | CR |
| U_americanus_GU724170 | *Ursus americanus* | GU724170 | modern | CR |
| U_americanus_GU724172 | *Ursus americanus* | GU724172 | modern | CR |
| U_americanus_GU724181 | *Ursus americanus* | GU724181 | modern | CR |
| U_americanus_GU724191 | *Ursus americanus* | GU724191 | modern | CR |
| U_americanus_GU724183 | *Ursus americanus* | GU724183 | modern | CR |
| U_americanus_GU724180 | *Ursus americanus* | GU724180 | modern | CR |
| U_americanus_GU724159 | *Ursus americanus* | GU724159 | modern | CR |
| U_americanus_GU724186 | *Ursus americanus* | GU724186 | modern | CR |
| U_americanus_GU724190 | *Ursus americanus* | GU724190 | modern | CR |
| U_americanus_GU724184 | *Ursus americanus* | GU724184 | modern | CR |
| U_americanus_GU724167 | *Ursus americanus* | GU724167 | modern | CR |
| U_americanus_GU724168 | *Ursus americanus* | GU724168 | modern | CR |
| U_americanus_GU724187 | *Ursus americanus* | GU724187 | modern | CR |
| U_americanus_GU724188 | *Ursus americanus* | GU724188 | modern | CR |
| U_americanus_GU724189 | *Ursus americanus* | GU724189 | modern | CR |
| U_americanus_GU724163 | *Ursus americanus* | GU724163 | modern | CR |
| U_americanus_GU724164 | *Ursus americanus* | GU724164 | modern | CR |
| U_americanus_GU724169 | *Ursus americanus* | GU724169 | modern | CR |
| U_americanus_GU724193 | *Ursus americanus* | GU724193 | modern | CR |
| U_americanus_GU724165 | *Ursus americanus* | GU724165 | modern | CR |
| U_americanus_X75863 | *Ursus americanus* | X75863 | modern | CR |
| U_americanus_AF305497 | *Ursus americanus* | AF305497 | modern | tRNA_Pro+CR |
| U_americanus_HE657193 | *Ursus americanus* | HE657193 | modern | CR |
| U_americanus_HE657194 | *Ursus americanus* | HE657194 | modern | CR |
| U_americanus_HE657195 | *Ursus americanus* | HE657195 | modern | CR |
| U_americanus_HE657196 | *Ursus americanus* | HE657196 | modern | CR |
| U_americanus_HE657197 | *Ursus americanus* | HE657197 | modern | CR |
| U_americanus_HE657198 | *Ursus americanus* | HE657198 | modern | CR |
| U_spelaeus_NC011112 | *Ursus spelaeus* | NC011112 | 44160 (+1400, -1190) | mitogenome |
| U_spelaeus_EU327344 | *Ursus spelaeus* | EU327344 | 31870 (+300, -270) | mitogenome |
| U_deningeri_KF437625 | *Ursus deningeri* | KF437625.2 | ~409000 | mitogenome |
| U_arctos_U34271 | *Ursus arctos* | U34271 | modern | cytB |
| U_arctos_U18870 | *Ursus arctos* | U18870 | modern | cytB |
| U_arctos_U18873 | *Ursus arctos* | U18873 | modern | cytB |
| U_arctos_U18874 | *Ursus arctos* | U18874 | modern | cytB |
| U_arctos_U18878 | *Ursus arctos* | U18878 | modern | cytB |
| U_arctos_U18881 | *Ursus arctos* | U18881 | modern | cytB |
| U_arctos_U18882 | *Ursus arctos* | U18882 | modern | cytB |
| U_arctos_U18887 | *Ursus arctos* | U18887 | modern | cytB |
| U_arctos_U18895 | *Ursus arctos* | U18895 | modern | cytB |
| U_arctos_U18896 | *Ursus arctos* | U18896 | modern | cytB |
| U_arctos_U18897 | *Ursus arctos* | U18897 | modern | cytB |
| U_arctos_EU567090 | *Ursus arctos* | EU567090 | modern | cytB |
| U_arctos_EU567091 | *Ursus arctos* | EU567091 | modern | cytB |
| U_arctos_JF900158 | *Ursus arctos* | JF900158 | 12143±46 | CR |
| U_arctos_JF900159 | *Ursus arctos* | JF900159 | 10650±100 | CR |
| U_arctos_JF900160 | *Ursus arctos* | JF900160 | 11920±85 | CR |
| U_arctos_JF900162 | *Ursus arctos* | JF900162 | 26340±320 | CR |
| U_arctos_JF900163 | *Ursus arctos* | JF900163 | 33310±770 | CR |
| U_arctos_JF900164 | *Ursus arctos* | JF900164 | 37870±1270 | CR |
| U_arctos_JF900166 | *Ursus arctos* | JF900166 | 4520±37 | CR |
| U_arctos_JF900167 | *Ursus arctos* | JF900167 | 3517±31 | CR |
| U_arctos_JF900168 | *Ursus arctos* | JF900168 | 11460±57 | CR |
| U_arctos_JF900169 | *Ursus arctos* | JF900169 | 8719±48 | CR |
| U_arctos_JF900170 | *Ursus arctos* | JF900170 | 10495±51 | CR |
| U_arctos_JF900172 | *Ursus arctos* | JF900172 | 9946±53 | CR |
| U_arctos_JF900173 | *Ursus arctos* | JF900173 | 28390±177 | CR |
| U_arctos_JF900174 | *Ursus arctos* | JF900174 | 4136±37 | CR |
| U_arctos_JF900175 | *Ursus arctos* | JF900175 | 2956±33 | CR |
| U_arctos_X75871 | *Ursus arctos* | X75871 | modern | CR |
| U_arctos_X75868 | *Ursus arctos* | X75868 | modern | CR |
| U_arctos_X75865 | *Ursus arctos* | X75865 | modern | CR |
| U_arctos_X75864 | *Ursus arctos* | X75864 | modern | CR |
| U_arctos_AJ809334 | *Ursus arctos* | AJ809334 | 40020±1000 | CR |
| U_arctos_EU400183 | *Ursus arctos* | EU400183 | 82500±7500 | CR |
| U_arctos_EU400182 | *Ursus arctos* | EU400182 | 82500±7500 | CR |
| U_arctos_EU400181 | *Ursus arctos* | EU400181 | 82500±7500 | CR |
| U_arctos_EF488504 | *Ursus arctos* | EF488504 | 17440±425 | CR |
| U_arctos_EU400179 | *Ursus arctos* | EU400179 | 6325±50 | CR |
| U_arctos_EF488506 | *Ursus arctos* | EF488506 | 2250±50 | CR |
| U_arctos_EF488505 | *Ursus arctos* | EF488505 | 3445±40 | CR |
| U_arctos_EF488503 | *Ursus arctos* | EF488503 | 4624±45 | CR |
| U_arctos_EF488501 | *Ursus arctos* | EF488501 | 1665±35 | CR |
| U_arctos_EF488498 | *Ursus arctos* | EF488498 | 1770±35 | CR |
| U_arctos_EF488497 | *Ursus arctos* | EF488497 | 350±40 | CR |
| U_arctos_EF488496 | *Ursus arctos* | EF488496 | 4645±40 | CR |
| U_arctos_EF488495 | *Ursus arctos* | EF488495 | 1570±35 | CR |
| U_arctos_EF488494 | *Ursus arctos* | EF488494 | 6525±50 | CR |
| U_arctos_EF488493 | *Ursus arctos* | EF488493 | 1750±30 | CR |
| U_arctos_EF488492 | *Ursus arctos* | EF488492 | 3845±40 | CR |
| U_arctos_EF488488 | *Ursus arctos* | EF488488 | 16440±65 | CR |
| U_arctos_AY796011 | *Ursus arctos* | AY796011 | 26310 | CR |
| U_arctos_AY082845 | *Ursus arctos* | AY082845 | 8000 | CR |
| U_arctos_AY082844 | *Ursus arctos* | AY082844 | 9995±95 | CR |
| U_arctos_AY082843 | *Ursus arctos* | AY082843 | 90-40Ky | CR |
| U_arctos_AY082842 | *Ursus arctos* | AY082842 | 36500±1150 | CR |
| U_arctos_AY082841 | *Ursus arctos* | AY082841 | 48164±3224 | CR |
| U_arctos_AY082840 | *Ursus arctos* | AY082840 | 19360±140 | CR |
| U_arctos_AY082839 | *Ursus arctos* | AY082839 | 35970±660 | CR |
| U_arctos_AY082837 | *Ursus arctos* | AY082837 | 42600±850 | CR |
| U_arctos_AY082836 | *Ursus arctos* | AY082836 | 41787±212 | CR |
| U_arctos_AY082835 | *Ursus arctos* | AY082835 | ˃59000 | CR |
| U_arctos_AY082834 | *Ursus arctos* | AY082834 | ˃56900 | CR |
| U_arctos_AY082833 | *Ursus arctos* | AY082833 | 47100±3100 | CR |
| U_arctos_AY082832 | *Ursus arctos* | AY082832 | 36137±783 | CR |
| U_arctos_AY082831 | *Ursus arctos* | AY082831 | ˃53900 | CR |
| U_arctos_AY082830 | *Ursus arctos* | AY082830 | 50800±1900 | CR |
| U_arctos_AY082829 | *Ursus arctos* | AY082829 | 20820±120 | CR |
| U_arctos_AY082828 | *Ursus arctos* | AY082828 | 15370±60 | CR |
| U_arctos_AY082827 | *Ursus arctos* | AY082827 | 12441±75 | CR |
| U_arctos_AY082824 | *Ursus arctos* | AY082824 | 12320±90 | CR |
| U_arctos_AY082823 | *Ursus arctos* | AY082823 | 15830±100 | CR |
| U_arctos_AY082816 | *Ursus arctos* | AY082816 | 10015±62 | CR |
| U_arctos_AY082813 | *Ursus arctos* | AY082813 | 14310±100 | CR |
| U_arctos_AY082812 | *Ursus arctos* | AY082812 | 11940±100 | CR |
| U_arctos_AY082810 | *Ursus arctos* | AY082810 | 20080±160 | CR |
| U_arctos_DQ914410 | *Ursus arctos* | DQ914410 | modern | CR |
| U_arctos_DQ914409 | *Ursus arctos* | DQ914409 | modern | CR |
| U_arctos_DQ914408 | *Ursus arctos* | DQ914408 | modern | CR |
| U_arctos_DQ914407 | *Ursus arctos* | DQ914407 | modern | CR |
| U_arctos_DQ914295 | *Ursus arctos* | DQ914295 | undated | CR |
| U_arctos_DQ914311 | *Ursus arctos* | DQ914311 | 87 | CR |
| U_arctos_DQ914332 | *Ursus arctos* | DQ914332 | 91 | CR |
| U_arctos_DQ914338 | *Ursus arctos* | DQ914338 | 84 | CR |
| U_arctos_DQ914348 | *Ursus arctos* | DQ914348 | 92 | CR |
| U_arctos_DQ914355 | *Ursus arctos* | DQ914355 | 92 | CR |
| U_arctos_DQ914356 | *Ursus arctos* | DQ914356 | 99 | CR |
| U_arctos_DQ914362 | *Ursus arctos* | DQ914362 | undated | CR |
| U_arctos_DQ914366 | *Ursus arctos* | DQ914366 | 101 | CR |
| U_arctos_DQ914367 | *Ursus arctos* | DQ914367 | 109 | CR |
| U_arctos_DQ914382 | *Ursus arctos* | DQ914382 | 86 | CR |
| U_arctos_DQ914389 | *Ursus arctos* | DQ914389 | 61 | CR |
| U_arctos_DQ914398 | *Ursus arctos* | DQ914398 | 100 | CR |
| U_arctos_DQ914399 | *Ursus arctos* | DQ914399 | undated | CR |
| U_arctos_DQ914400 | *Ursus arctos* | DQ914400 | 147 | CR |
| U_arctos_DQ914403 | *Ursus arctos* | DQ914403 | 54 | CR |
| U_arctos_DQ914404 | *Ursus arctos* | DQ914404 | 53 | CR |
| U_arctos_DQ914405 | *Ursus arctos* | DQ914405 | 57 | CR |
| U_arctos_DQ914406 | *Ursus arctos* | DQ914406 | modern | CR |
| U_arctos_DQ914411 | *Ursus arctos* | DQ914411 | modern | CR |
| U_arctos_EF033706 | *Ursus arctos* | EF033706 | modern | CR |
| U_arctos_EF033710 | *Ursus arctos* | EF033710 | modern | CR |
| U_arctos_EF033712 | *Ursus arctos* | EF033712 | modern | CR |
| U_arctos_EF033719 | *Ursus arctos* | EF033719 | modern | CR |
| U_arctos_EF033731 | *Ursus arctos* | EF033731 | modern | CR |
| U_arctos_EF033733 | *Ursus arctos* | EF033733 | modern | CR |
| U_arctos_EF033734 | *Ursus arctos* | EF033734 | modern | CR |
| U_arctos_EF033737 | *Ursus arctos* | EF033737 | modern | CR |
| U_arctos_EF033738 | *Ursus arctos* | EF033738 | modern | CR |
| U_arctos_EF033826 | *Ursus arctos* | EF033826 | modern | CR |
| U_arctos_EF033845 | *Ursus arctos* | EF033845 | modern | CR |
| U_arctos_EF033855 | *Ursus arctos* | EF033855 | modern | CR |
| U_arctos_EF033858 | *Ursus arctos* | EF033858 | modern | CR |
| U_arctos_EF033860 | *Ursus arctos* | EF033860 | modern | CR |
| U_arctos_EF033869 | *Ursus arctos* | EF033869 | modern | CR |
| U_arctos_EF033874 | *Ursus arctos* | EF033874 | modern | CR |
| U_arctos_EF033875 | *Ursus arctos* | EF033875 | modern | CR |
| U_arctos_EF033878 | *Ursus arctos* | EF033878 | modern | CR |
| U_arctos_EF033890 | *Ursus arctos* | EF033890 | modern | CR |
| U_arctos_EF033896 | *Ursus arctos* | EF033896 | modern | CR |
| U_arctos_EF033897 | *Ursus arctos* | EF033897 | modern | CR |
| U_arctos_EF033916 | *Ursus arctos* | EF033916 | modern | CR |
| U_arctos_EF033999 | *Ursus arctos* | EF033999 | modern | CR |
| U_arctos_EF034002 | *Ursus arctos* | EF034002 | modern | CR |
| U_arctos_EF034017 | *Ursus arctos* | EF034017 | modern | CR |
| U_arctos_EF034022 | *Ursus arctos* | EF034022 | modern | CR |
| U_arctos_EF034023 | *Ursus arctos* | EF034023 | modern | CR |
| U_arctos_EF034025 | *Ursus arctos* | EF034025 | modern | CR |
| U_arctos_AB010727 | *Ursus arctos* | AB010727 | modern | CR |
| U_arctos_AB010728 | *Ursus arctos* | AB010728 | modern | CR |
| U_arctos_JQ823238 | *Ursus arctos* | JQ823238 | 5310 ± 20 | CR |
| U_arctos_JQ823242 | *Ursus arctos* | JQ823242 | 6210 ± 100 | CR |
| U_arctos_HE657216 | *Ursus arctos* | HE657216 | modern | CR |
| U_arctos_HE657214 | *Ursus arctos* | HE657214 | modern | CR |
| U_arctos_HE657213 | *Ursus arctos* | HE657213 | modern | CR |
| U_arctos_HE657212 | *Ursus arctos* | HE657212 | modern | CR |
| U_arctos_HE657211 | *Ursus arctos* | HE657211 | modern | CR |
| U_arctos_HE657210 | *Ursus arctos* | HE657210 | modern | CR |
| U_arctos_HE657207 | *Ursus arctos* | HE657207 | modern | CR |
| U_arctos_HE657205 | *Ursus arctos* | HE657205 | modern | CR |
| U_arctos_AM411397 | *Ursus arctos* | AM411397 | 1680 ± 35 | CR+cytB |
| U_arctos_AM411399 | *Ursus arctos* | AM411399 | 1285 ± 60 | CR+cytB |
| U_arctos_AM411403 | *Ursus arctos* | AM411403 | 7345 ± 40 | CR+cytB |
| U_arctos_FN292971 | *Ursus arctos* | FN292971 | 145 | CR+cytB |
| U_arctos_FN292973 | *Ursus arctos* | FN292973 | 125 | CR+cytB |
| U_arctos_FN292977 | *Ursus arctos* | FN292977 | modern | CR+cytB |
| U_arctos_FN292979 | *Ursus arctos* | FN292979 | modern | CR+cytB |
| U_arctos_FN292982 | *Ursus arctos* | FN292982 | modern | CR+cytB |
| U_arctos_FN292975 | *Ursus arctos* | FN292975 | 40 | CR+cytB |
| U_arctos_FN292974 | *Ursus arctos* | FN292974 | 45 | CR+cytB |
| U_arctos_GU573489 | *Ursus arctos* | GU573489 | modern | mitogenome |
| U_arctos_GU573487 | *Ursus arctos* | GU573487 | modern | mitogenome |
| U_arctos_GU573486 | *Ursus arctos* | GU573486 | modern | mitogenome |
| U_arctos_JX196369 | *Ursus arctos* | JX196369 | modern | mitogenome |
| U_arctos_JX196368 | *Ursus arctos* | JX196368 | modern | mitogenome |
| U_arctos_JX196367 | *Ursus arctos* | JX196367 | modern | mitogenome |
| U_arctos_GU573491 | *Ursus arctos* | GU573491 | modern | mitogenome |
| U_arctos_AF303110 | *Ursus arctos* | AF303110 | modern | mitogenome |
| U_arctos_EU497665 | *Ursus arctos* | EU497665 | modern | mitogenome |
| U_arctos_AP012593 | *Ursus arctos* | AP012593 | modern | mitogenome |
| U_arctos_AP012592 | *Ursus arctos* | AP012592 | modern | mitogenome |
| U_arctos_AP012591 | *Ursus arctos* | AP012591 | modern | mitogenome |
| U_arctos_AP012590 | *Ursus arctos* | AP012590 | modern | mitogenome |
| U_arctos_AP012589 | *Ursus arctos* | AP012589 | modern | mitogenome |
| U_arctos_AP012588 | *Ursus arctos* | AP012588 | modern | mitogenome |
| U_arctos_AP012586 | *Ursus arctos* | AP012586 | modern | mitogenome |
| U_arctos_AP012585 | *Ursus arctos* | AP012585 | modern | mitogenome |
| U_arctos_AP012584 | *Ursus arctos* | AP012584 | modern | mitogenome |
| U_arctos_AP012581 | *Ursus arctos* | AP012581 | modern | mitogenome |
| U_arctos_AP012580 | *Ursus arctos* | AP012580 | modern | mitogenome |
| U_arctos_AP012579 | *Ursus arctos* | AP012579 | modern | mitogenome |
| U_arctos_AP012578 | *Ursus arctos* | AP012578 | modern | mitogenome |
| U_arctos_AP012577 | *Ursus arctos* | AP012577 | modern | mitogenome |
| U_arctos_AP012576 | *Ursus arctos* | AP012576 | modern | mitogenome |
| U_arctos_AP012575 | *Ursus arctos* | AP012575 | modern | mitogenome |
| U_arctos_AP012574 | *Ursus arctos* | AP012574 | modern | mitogenome |
| U_arctos_AP012573 | *Ursus arctos* | AP012573 | modern | mitogenome |
| U_arctos_AP012572 | *Ursus arctos* | AP012572 | modern | mitogenome |
| U_arctos_AP012571 | *Ursus arctos* | AP012571 | modern | mitogenome |
| U_arctos_AP012570 | *Ursus arctos* | AP012570 | modern | mitogenome |
| U_arctos_AP012569 | *Ursus arctos* | AP012569 | modern | mitogenome |
| U_arctos_AP012568 | *Ursus arctos* | AP012568 | modern | mitogenome |
| U_arctos_AP012567 | *Ursus arctos* | AP012567 | modern | mitogenome |
| U_arctos_AP012566 | *Ursus arctos* | AP012566 | modern | mitogenome |
| U_arctos_AP012565 | *Ursus arctos* | AP012565 | modern | mitogenome |
| U_arctos_AP012564 | *Ursus arctos* | AP012564 | modern | mitogenome |
| U_arctos_AP012563 | *Ursus arctos* | AP012563 | modern | mitogenome |
| U_arctos_AP012562 | *Ursus arctos* | AP012562 | modern | mitogenome |
| U_arctos_HQ685964 | *Ursus arctos* | HQ685964 | modern | mitogenome |
| U_arctos_HQ685957 | *Ursus arctos* | HQ685957 | modern | mitogenome |
| U_arctos_HQ685942 | *Ursus arctos* | HQ685942 | modern | mitogenome |
| U_arctos_HQ685927 | *Ursus arctos* | HQ685927 | modern | mitogenome |
| U_arctos_HQ685911 | *Ursus arctos* | HQ685911 | modern | mitogenome |
| U_arctos_KX641336 | *Ursus arctos* | KX641336 | 36680 | mitogenome |
| U_arctos_KX641328 | *Ursus arctos* | KX641328 | 9045 | mitogenome |
| U_arctos_KX641327 | *Ursus arctos* | KX641327 | 9240 | mitogenome |
| U_arctos_KX641326 | *Ursus arctos* | KX641326 | 7340 | mitogenome |
| U_arctos_KX641325 | *Ursus arctos* | KX641325 | 6750 | mitogenome |
| U_arctos_KX641323 | *Ursus arctos* | KX641323 | 4160 | mitogenome |
| U_arctos_KX641322 | *Ursus arctos* | KX641322 | 4115 | mitogenome |
| U_arctos_KX641319 | *Ursus arctos* | KX641319 | 8720 | mitogenome |
| U_maritimus_HM584820 | *Ursus maritimus* | HM584820 | 115000 | CR |
| U_maritimus_EF033727 | *Ursus maritimus* | EF033727 | modern | CR |
| U_maritimus_EF033728 | *Ursus maritimus* | EF033728 | modern | CR |
| U_maritimus_EF033729 | *Ursus maritimus* | EF033729 | modern | CR |
| U_maritimus_EF033730 | *Ursus maritimus* | EF033730 | modern | CR |
| U_maritimus_JF900105 | *Ursus maritimus* | JF900105 | modern | CR |
| U_maritimus_EU567096 | *Ursus maritimus* | EU567096 | modern | cytB |
| U_maritimus_JF900107 | *Ursus maritimus* | JF900107 | modern | CR |
| U_maritimus_JF900109 | *Ursus maritimus* | JF900109 | modern | CR |
| U_maritimus_JF900110 | *Ursus maritimus* | JF900110 | modern | CR |
| U_maritimus_JF900112 | *Ursus maritimus* | JF900112 | modern | CR |
| U_maritimus_JF900116 | *Ursus maritimus* | JF900116 | modern | CR |
| U_maritimus_JF900121 | *Ursus maritimus* | JF900121 | 80 | CR |
| U_maritimus_JF900133 | *Ursus maritimus* | JF900133 | modern | CR |
| U_maritimus_JF900135 | *Ursus maritimus* | JF900135 | 81 | CR |
| U_maritimus_JF900151 | *Ursus maritimus* | JF900151 | 77 | CR |
| U_maritimus_JF900122 | *Ursus maritimus* | JF900122 | 111 | CR |
| U_maritimus_JF900123 | *Ursus maritimus* | JF900123 | 76 | CR |
| U_maritimus_JF900124 | *Ursus maritimus* | JF900124 | 76 | CR |
| U_maritimus_JF900134 | *Ursus maritimus* | JF900134 | 111 | CR |
| U_maritimus_JF900139 | *Ursus maritimus* | JF900139 | historic | CR |
| U_maritimus_JF900125 | *Ursus maritimus* | JF900125 | 79 | CR |
| U_maritimus_JF900138 | *Ursus maritimus* | JF900138 | 81 | CR |
| U_maritimus_GU573488 | *Ursus maritimus* | GU573488 | 120000 | mitogenome |
| U_maritimus_GU573490 | *Ursus maritimus* | GU573490 | modern | mitogenome |
| U_maritimus_GU573485 | *Ursus maritimus* | GU573485 | modern | mitogenome |
| U_maritimus_JX196374 | *Ursus maritimus* | JX196374 | modern | mitogenome |
| U_maritimus_JX196373 | *Ursus maritimus* | JX196373 | modern | mitogenome |
| U_maritimus_JX196372 | *Ursus maritimus* | JX196372 | modern | mitogenome |
| U_maritimus_JX196371 | *Ursus maritimus* | JX196371 | modern | mitogenome |
| U_maritimus_JX196370 | *Ursus maritimus* | JX196370 | modern | mitogenome |
| U_maritimus_JX196392 | *Ursus maritimus* | JX196392 | modern | mitogenome |
| U_maritimus_JX196391 | *Ursus maritimus* | JX196391 | modern | mitogenome |
| U_maritimus_JX196389 | *Ursus maritimus* | JX196389 | modern | mitogenome |
| U_maritimus_JX196388 | *Ursus maritimus* | JX196388 | modern | mitogenome |
| U_maritimus_JX196387 | *Ursus maritimus* | JX196387 | modern | mitogenome |
| U_maritimus_JX196386 | *Ursus maritimus* | JX196386 | modern | mitogenome |
| U_maritimus_JX196385 | *Ursus maritimus* | JX196385 | modern | mitogenome |
| U_maritimus_JX196384 | *Ursus maritimus* | JX196384 | modern | mitogenome |
| U_maritimus_JX196383 | *Ursus maritimus* | JX196383 | modern | mitogenome |
| U_maritimus_JX196382 | *Ursus maritimus* | JX196382 | modern | mitogenome |
| U_maritimus_JX196381 | *Ursus maritimus* | JX196381 | modern | mitogenome |
| U_maritimus_JX196380 | *Ursus maritimus* | JX196380 | modern | mitogenome |
| U_maritimus_JX196379 | *Ursus maritimus* | JX196379 | modern | mitogenome |
| U_maritimus_JX196378 | *Ursus maritimus* | JX196378 | modern | mitogenome |
| U_maritimus_JX196377 | *Ursus maritimus* | JX196377 | modern | mitogenome |
| U_maritimus_JX196376 | *Ursus maritimus* | JX196376 | modern | mitogenome |
| U_maritimus_JX196375 | *Ursus maritimus* | JX196375 | modern | mitogenome |
| U_maritimus_AP012597 | *Ursus maritimus* | AP012597 | modern | mitogenome |
| U_maritimus_AP012596 | *Ursus maritimus* | AP012596 | modern | mitogenome |
| U_maritimus_AP012595 | *Ursus maritimus* | AP012595 | modern | mitogenome |
| U_maritimus_AP012594 | *Ursus maritimus* | AP012594 | modern | mitogenome |

**References**

Melton, T. & Holland, C. 2007 Routine Forensic Use of the Mitochondrial 12S Ribosomal RNA Gene for Species Identification. *Journal Forensic Sci.* **52**, 1305-1307. (doi:10.1111/j.1556-4029.2007.00553.x).

Taberlet, P. & Bouvet, J. 1994 Mitochondrial DNA polymorphism, phylogeography, and conservation genetics of the brown bear *Ursus arctos* in Europe. *Proc. R. Soc. B* **255**, 195-200. (doi:10.1098/rspb.1994.0028).

Rohland, N., Siedel, H. & Hofreiter, M. 2004 Nondestructive DNA extraction method for mitochondrial DNA analyses of museum specimens. *BioTechniques* **36**, 814-821.
