# Supplementary figures and images for: Evolutionary history of enigmatic bears in the Tibetan Plateau–Himalaya region and the identity of the yeti
Source: Proc Biol Sci. 2017 Nov 29;284(1868):20171804. doi: 10.1098/rspb.2017.1804 (PMC5740279; doi:10.1098/rspb.2017.1804)

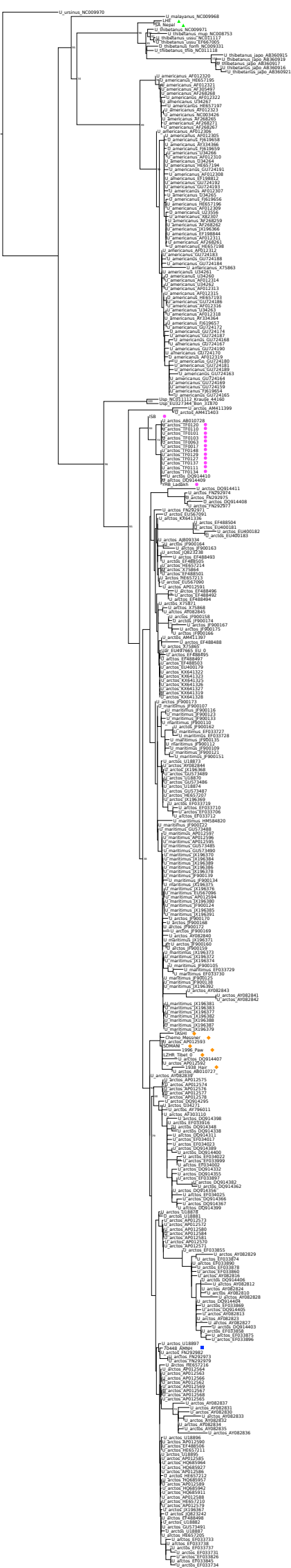

Supplement: Supplementary Figure S1 [file rspb20171804supp2.pdf]

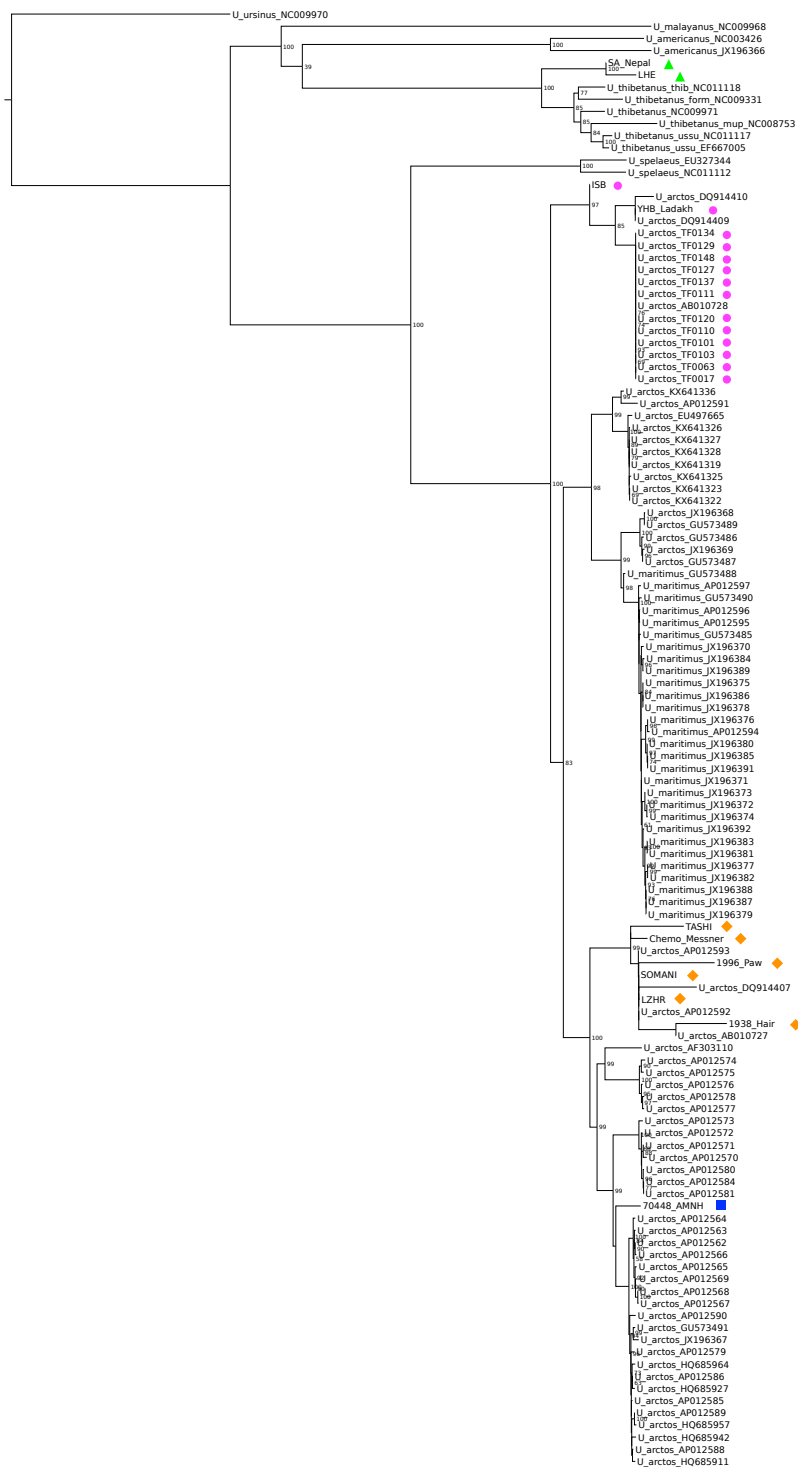

Supplement: Supplementary Figure S2 [file rspb20171804supp3.pdf]

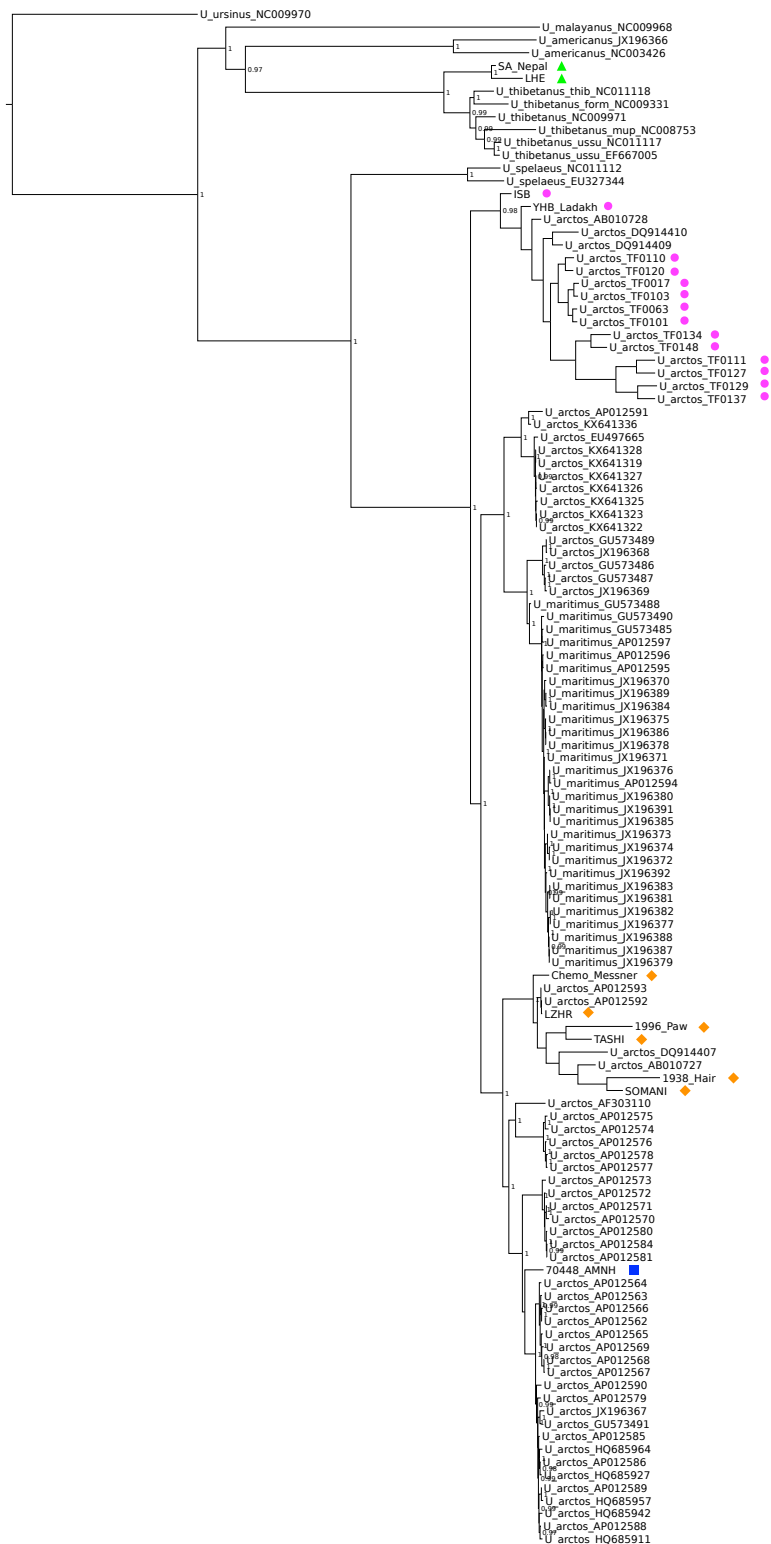

Supplement: Supplementary Figure S3 [file rspb20171804supp4.pdf]

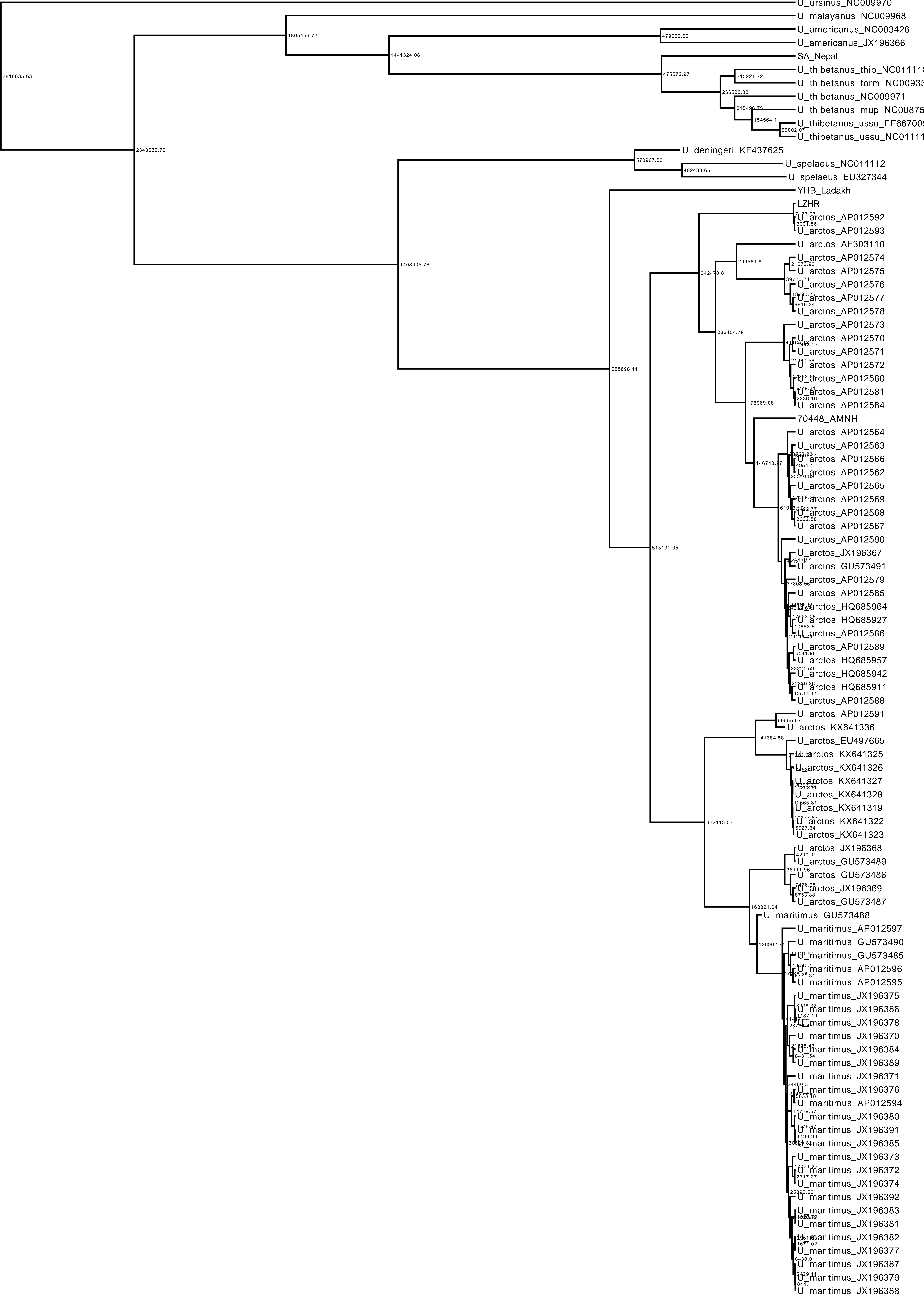

300000.0

Supplement: Supplementary Figure S4 [file rspb20171804supp5.pdf]
